# The Effectiveness of Web-Based Interventions to Promote Health Behaviour Change in Adolescents: A Systematic Review

**DOI:** 10.3390/nu14061258

**Published:** 2022-03-16

**Authors:** Daniela de Sousa, Adriana Fogel, José Azevedo, Patrícia Padrão

**Affiliations:** 1 EPIUnit—Instituto de Saúde Pública, Universidade do Porto, 4050-600 Porto, Portugal; daniela.sousa@ispup.up.pt (D.d.S.); adriana.fogel@gmail.com (A.F.); azevedo@letras.up.pt (J.A.); 2 Laboratório para a Investigação Integrativa e Translacional em Saúde Populacional (ITR), 4050-600 Porto, Portugal; 3 Faculdade de Letras, Universidade do Porto, 4150-564 Porto, Portugal; 4 Faculdade de Ciências da Nutrição e Alimentação, Universidade do Porto, 4150-180 Porto, Portugal

**Keywords:** systematic review, web-based intervention, health behaviour, behaviour change, adolescents

## Abstract

Although web-based interventions are attractive to researchers and users, the evidence about their effectiveness in the promotion of health behaviour change is still limited. Our aim was to review the effectiveness of web-based interventions used in health behavioural change in adolescents regarding physical activity, eating habits, tobacco and alcohol use, sexual behaviour, and quality of sleep. Studies published from 2016 till the search was run (May-to-June 2021) were included if they were experimental or quasi-experimental studies, pre-post-test studies, clinical trials, or randomized controlled trials evaluating the effectiveness of web-based intervention in promoting behaviour change in adolescents regarding those health behaviours. The risk of bias assessment was performed by using the Effective Public Health Practice Project (EPHPP)—Quality Assessment Tool for Quantitative Studies. Fourteen studies were included. Most were in a school setting, non-probabilistic and relatively small samples. All had a short length of follow-up and were theory driven. Thirteen showed significant positive findings to support web-based interventions’ effectiveness in promoting health behaviour change among adolescents but were classified as low evidence quality. Although this review shows that web-based interventions may contribute to health behaviour change among adolescents, these findings rely on low-quality evidence, so it is urgent to test these interventions in larger controlled trials with long-term maintenance.

## 1. Introduction

A major concern to public health researchers is lifestyle behaviours. Risky behaviours, such as tobacco and alcohol use, unhealthy food habits, physical inactivity, risky sexual practices, and insufficient sleep duration, play a significant role in many of the leading causes of death worldwide [1]. According to data from 2015, 70% of all preventable deaths from non-communicable diseases in adults are related to lifestyle risk factors adopted during adolescence [2].

Adolescence is a critical period since many unhealthy habits and risky behaviours begin at this age. However, it is also a window of opportunity for the development of health-protective behaviours since health-related habits adopted at this age tend to persist into adulthood [3]. For this reason, it is suggested that public health interventions aimed to prevent or stop risky behaviours should target this life period [4], knowing that improving adolescents’ health now is also ensuring a better future for the next generations [5,6].

A positive aspect for health promotion is that, since behaviours are modifiable health-related variables, the threat they represent is greatly preventable [7,8,9] and even minor changes in human behaviours can improve the overall population’s health [1]. However, the drawback is that behaviour change is a very complex and iterative process, in which even individuals who are aware of better health practices still fall short in adjusting their behaviour [10].

Therefore, focusing on achieving and maintaining successful behavioural change in individuals and communities is a key question for health research [10]. Over the years, theories, models, and techniques have been suggested to understand and predict behaviour [11], contributing to the support of planners in handling challenges in the research concept, implementation, and evaluation to improve the effectiveness of behavioural interventions [12].

Until this moment, there has been no consensus on the key role that these behavioural interventions can play in population-level health. However, there is already agreement that understanding theories of behaviour change is an essential element of successful health-related interventions [13].

The relationship between the healthcare provider and the patient is now much more different than it was before, and at the end of the 20th century, researchers and health professionals began to realize how important shared decision-making is in healthcare service. Additionally, a growing interest in participatory approaches to health promotion has been observed, especially in interventions targeting children and adolescents. So, as the medical paradigm was changing, digital technologies able to raise patient empowerment were also becoming more readily available [14].

Thus, digital health promotion interventions, especially internet-based technologies, have been suggested as important tools to improve individuals’ health and the quality of healthcare services and to reduce health inequalities due to their large-scale availability. Although there is a lack of robust evidence to support the effectiveness of web-based interventions, it seems to be a promising approach to support behavioural health change [15], which is becoming increasingly attractive to researchers [16].

**Figure 1:**
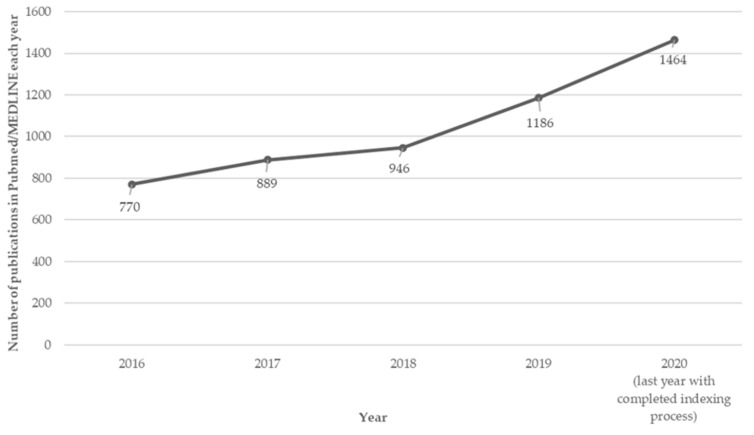
Self-elaborated graph of trends by year of publication for search terms “health AND web-based intervention” from PubMed/MEDLINE data on 26 August 2021.

The interest in using web-based interventions in the health field is still growing. The number of publications reveals this interest since this amount increased from 770 in 2016 to 1464 in 2020. In this same period, there was an increase of 683% in Pubmed/MEDLINE publication results for “health AND web-based intervention” (Figure 1).

Although web-based interventions may be attractive to both users and researchers, it is crucial to summarize evidence about their effectiveness. It is essential to identify which are the delivery modes and the behavioural change theories and techniques most frequently used to promote positive health behavioural change and its maintenance in the long term [13].

Since there is a wide range of digital technologies (e.g., social media, telemedicine, data analytics, artificial intelligence, personalized medicine, wearables, mobile apps, electronic medical records, web-resources for health education, among others) and each of them has unique capabilities and specificities [17], characterizing each one individually will help researchers construct more robust evidence to better explain their impact on different health outcomes.

So, it is valuable to summarize the recent findings concerning the effectiveness of web-based interventions in adolescents’ health, especially those more critical to the 10–24-year-old age range, namely physical activity, eating, smoking, alcohol use, sexual behaviour, and quality of sleep. Additionally, it is important to evaluate how these interventions are modelled and used to support and maintain behavioural health change in this population. The main objective of this study is to respond to this need by systematically reviewing the literature published in the last 5 years.

## 2. Methods

### 2.1. Data Sources

This systematic review was prepared in accordance with the Preferred Reporting Items for Systematic Reviews and Meta-Analyses (PRISMA) statement [18]. The review protocol was registered on the International Prospective Register of Systematic Reviews (PROSPERO) (registration number CRD42021275508).

The electronic databases of PubMed/MEDLINE, Scopus, APA Journals, Web of Science and SAGE Journal were searched from May to June 2021. The search terms used were organized in three main sections: population, intervention, and health outcomes, each illustrated by keywords and synonyms. All the terms used in the search strategy were connected by the Boolean operator AND, between each main section, and OR, within each section, as detailed in Table S1. We set the alerts for each database to reach new added results. Additional articles were identified from the reference list of retrieved articles by applying a reverse snowballing search.

### 2.2. Inclusion and Exclusion Criteria

Studies were eligible for inclusion if their full text was published in scientific journals in English, Portuguese, or Spanish. Only studies published from 2016 till June of 2021 were included, considering the wide range of digital technologies [17], the raising of patient empowerment and their relationship with technology [14], as well as the exponential and rapid development of digital technology in this last five-year period [19].

Considering the PICO-S framework (population, intervention, comparison, outcomes, study design), we defined the other inclusion and exclusion criteria that we described below. Articles that cover all inclusion criteria were considered in this systematic review.

#### 2.2.1. Participants/Population

##### Inclusion Criteria

The population included only adolescents according to the definition used by Sawyer et al., (2018), which corresponds to people between 10 and 24 years old [20] and who have participated in web-based health interventions.

##### Exclusion Criteria

Interventions targeted not directly to participants aged 10 to 24 years old but targeted their parents, educators, or healthcare professionals were excluded. Interventions targeting multiple ages were excluded if it was not possible to isolate the desired target age group.

Specific subgroups such as disabled people or people with irreversible clinical conditions or major chronic diseases were excluded since the review focused on general adolescents.

#### 2.2.2. Intervention/Exposure

##### Inclusion Criteria

In our review, we considered the definition described by Barak et al., (2009), which mentions that a web-based intervention is “a primarily self-guided intervention programme that is executed using a prescriptive online programme operated through a website and used by consumers seeking health and mental-health related assistance. The intervention programme itself attempts to create positive change and or improve/enhance knowledge, awareness, and understanding via the provision of sound health-related material and use of interactive Web-based components” [21]. We considered web-based interventions developed for health promotion to improve and/or maintain positive health behaviours. To be included, the web-based intervention can be a stand-alone intervention or a multicomponent intervention where the use of web-based resources is one of the intervention’s components.

##### Exclusion Criteria

We excluded articles if they do not report a web-based intervention, if the intervention does not aim to reduce lifestyle risk factors (non-health promotion interventions) or if the interventions aim to improve disease screening or to control major chronic diseases.

#### 2.2.3. Comparator(s)/Control

##### Inclusion Criteria

Studies that compare the proposed intervention/exposure to another intervention or a non-intervention group, as well as studies with a pre-test and post-test design, were considered in this review.

##### Exclusion Criteria

Studies with no control group and only with a post-test design were excluded.

#### 2.2.4. Outcome(s)

##### Inclusion Criteria

Regarding the main objective of this systematic review, we included studies if they analysed the effectiveness of the intervention to promote desired behavioural change using quantitative or mixed methods. This may include outcomes such as the extent and maintenance of behaviour changes, risk reduction, cognitions and attitudes, behavioural intention, subjective norm, self-efficacy, perceived behavioural control and pre-conditions for practising and maintaining the desired behaviours (regarding physical activity, eating habits and weight control, smoking, alcohol use, sexual behaviour, and quality of sleep).

Secondary outcomes such as adoption and adherence rates of the web-based intervention, patient-reported experience, feasibility and usability assessments, and coherence of the technology with behavioural change techniques will also be analysed when available.

##### Exclusion Criteria

Articles only evaluating the effectiveness of the intervention by qualitative methods were excluded, as well as those that did not have a measurement of the effectiveness. Additionally, studies not evaluating health outcomes related at least to one of these health behaviours were excluded: physical activity, eating habits and weight control, smoking, alcohol use, sexual behaviour, and quality of sleep.

#### 2.2.5. Study Design

##### Inclusion Criteria

The final set of included studies was limited to quantitative or mixed methods studies as experimental studies, quasi-experimental studies, before-and-after studies/pre-post-test studies, clinical trials, and randomized control trials.

##### Exclusion Criteria

Other types of publications, such as case studies, systematic reviews, meta-analyses, case reports and series, ideas, editorials, opinions, study protocols and studies using only qualitative methods were not included.

### 2.3. Data Extraction

The main author (DS) performed a search of the electronic databases. The articles found by databases search, after applying the filters, were imported into Endnote TM20 software and the duplicate records were removed by automation tools and manual search. Early screening by titles and abstracts was performed by one author (DS) based on the aim of the study and the eligibility criteria. Those articles identified as being potentially eligible were fully examined by two researchers (DS and AF) separately to make sure they met the inclusion criteria. In case of discrepancies, the decision was discussed and deliberated by both reviewers. If the disagreement persisted, it was solved by two other authors (PP and JA).

The articles that met the specified inclusion criteria had their data extracted by the main reviewer (DS) and validated by a second reviewer (AF) using a table developed in Microsoft^®^ Excel by the study team (Table S2).

Two researchers (DS and AF) independently performed the risk of bias assessment for all included studies using the Effective Public Health Practice Project—Quality Assessment Tool for Quantitative Studies (EPHPP), which has been validated for use in public health research [22]. Even though we considered several tools, we chose the Effective Public Health Practice Project (EPHPP) tool because it was a validated tool that could be used across multiple study designs and had been developed to be used in systematic reviews about effectiveness to questions related to public health programs [22,23].

The global rating for each article was assessed by evaluation as weak, moderate, or strong regarding six domain ratings: selection bias, study design, confounders, blinding, data collection methods, withdrawals, and drop-outs, according to a standardized guide and dictionary (Table 1). Those with no weak ratings and at least four strong ratings were considered strong. Those with less than four strong ratings and one weak rating were considered moderate. Finally, those with two or more weak ratings were considered weak. Two other domains were included in the assessment, but they were not included in the overall score (the integrity of the intervention and analysis) [23]. After classifying all dimensions, both reviewers (DS and AF) discussed and compared their assessments. When discrepancies occurred, the reason was identified as oversight, differences in interpretation of criteria or differences in interpretation of the study. After discussion, both researchers agreed on a final decision.

### 2.4. Data Synthesis

Considering the broad health behaviours included in our research question, substantial heterogeneity between studies was found regarding their aims, methods and reported outcomes. Thus, we decided to perform a qualitative synthesis to summarize the extracted data rather than perform a meta-analysis. By doing so, we intended to systematically review web-based interventions related to health behaviour change to interpret the results and draw conclusions about their effectiveness, feasibility, usability, and use of behaviour changing techniques. Furthermore, we also identified the limitations and proposed directions for future research.

## 3. Results

Table 2 encompasses the summary of narrative synthesis, and it includes authors, publication year, country/region, setting of the study, study design, health outcomes and main findings. More detailed information is available in Tables S3–S5.

### 3.1. Study Selection

**Figure 2:**
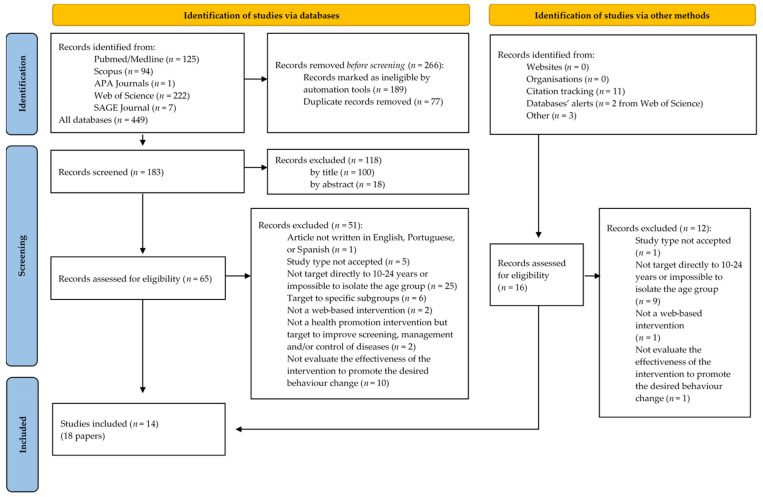
Study Flow Diagram adapted from: [18]. For more information, visit: http://www.prisma-statement.org/ (accessed on 29 January 2022).

As described in the PRISMA flowchart (Figure 2), 449 results were found through the search in the five electronic databases. Of those, 189 results were marked as ineligible by automated tools of the databases, the remaining articles were imported into Endnote TM20 software, and 77 results were found to be duplicate records. In total, 266 results were removed before the screening. One of the authors (DS) screened the title and abstracts of 183 studies and, based on the purpose of the study and the inclusion and exclusion criteria, DS identified 65 studies sought for retrieval. The full texts of those potentially eligible studies were independently assessed by two reviewers (DS and AF) using the inclusion and exclusion criteria. In cases of discrepancies, the decision was discussed and deliberated by both reviewers. If the disagreement persisted, it was solved by the two other authors (PP and JA).

The reference list of retrieved articles was searched, which resulted in 11 articles being added to be assessed for eligibility, two others were identified from databases’ alerts and three more were found by searching the intervention names of the articles excluded because they did not evaluate the effectiveness of the intervention. In total, 16 records were identified via other methods, and their full texts were compared with our eligibility criteria; of those, 12 were excluded.

In total, 18 records met all the inclusion criteria, describing a total of 14 different interventions. We found five records with the same main author describing the same intervention (The eCHECKUP TO GO) [24,25,26,27,28], so prevent duplicate studies that might lead to biased results, we assessed the time of recruitment, the sample size, and the time of follow-up [29]. The decision was to include in the narrative synthesis the work of Doumas, D. M. et al., (2021) since it had the best combination of the longest time of follow-up (6 months) with the greatest sample size (*n* = 311) [28].

### 3.2. Description of the Studies 

From among the 14 included studies, 6 were conducted in the United States of America (42.9%) [28,30,31,32,33,34], 3 in European countries (21.4%) [35,36,37], 3 in Asian countries (21.4%) [38,39,40] and 2 in Mexico (14.3%) [41,42]. Of those, 10 were implemented in an educational setting (71.4%) [28,30,32,33,37,38,39,40,41,42].

Concerning study design, seven studies were randomized controlled trials (50.0%) [28,32,33,35,36,37,40], four were quasi-experimental studies (28.6%) [38,39,41,42] and three had a pre and post-test design (21.4%) [30,31,34].

Regarding the desired behaviour change, four interventions intended to promote physical activity (28.6%) [30,31,38,39], one aimed to modify physical activity simultaneously with fruit and vegetable consumption (7.1%) [40], four were related to alcohol use (28.6%) [28,34,35,37], four tried to prevent risky sexual behaviours (28.6%) [33,36,41,42] and one proposed preventing tobacco use (7.1%) [32]. None of the sleep hygiene interventions records was found to meet the inclusion criteria.

**Table 2:** Summary of narrative synthesis of included studies.

Author, Year, Country	Setting	Study Design	Participants	Web-Based Intervention	Health Outcomes of Interest	Main Findings
Wilson, M. et al., (2017) [30] USA, North-western United States	School	One-group pre-/post-test design (pre-experimental)	*N* = 20 students (convenience sample) Mean age of 16.8 years.	Multicomponent Intervention: wearable digital tracking device using an Internet-based platform + group physical activities + nutrition group education/individual counselling session on healthy eating + weekly goal-setting sessions.	Measured at baseline and post-intervention: BMI calculation. Blood glucose level. Blood pressure and pulse measurements. Fitness and cardiovascular fitness. Cognitive and affective variables related to health behaviours. Adolescents’ physical activity (PA) and healthy eating. Self-efficacy for PA and healthy eating. Self-determination. Screen time.	Participants showed improvements from pre-test to post-test in health and fitness markers (positive changes in weight, fitness, and cardiovascular measurements) and improved motivation toward PA and reduced screen time.
Larsen, B. et al., (2018) [31] USA (San Diego, CA)	Hispanic community	Pre/post-test design (Single-arm pilot trial)	*N* = 21 Latina adolescents Mean age of 14.7 years.	Website mobile phone friendly (tailored Internet-delivered activity manuals, computer-expert system tailored reports, activity tip sheets, and a guide of local activity resources)	Measured at baseline and follow-up (12 weeks): PA by 7-day physical activity recall (PAR) interview and ActiGraph GT3X+ accelerometers.	Results from the 7-day PAR showed that positive changes in PA at 12 weeks were seen not just in quantity but also in type. The usage of validated self-report measures showed to be better than accelerometers among this population since there are some activities in which the accelerometer may not be worn or that were not well measured by the accelerometer.
Huang, S. J. et al., (2019) [39] Taiwan, Taipei City	School	Quasi-experimental (Three-armed)	*N* initial = 617 students Mean age of 11.4 years.	Two experimental groups: One using a web-based exercise program applying a self-management strategy combined with geographical information system (GIS) mapping function and using a narrative animated cartoon. The other was knowledge-only using only the animated story.	Measured at baseline, immediately post and 3-month follow-up: PA by the Chinese version of the Child/Adolescent Activity Log. Exercise-related self-efficacy using a 5-item Exercise-Related-Self-Efficacy Scale. Perceived benefit of PA using a self-developed 7-item Perceived-Benefit-of-Exercise Scale.	This intervention using self-management strategy + GIS mapping function was effective in producing small but significant increases in school children’s self-efficacy and PA. The perceived benefit and self-efficacy of regular PA might have partly affected the participants’ PA levels because the self-efficacy factor was always higher for both experimental groups than for the control at the post-test and follow-up; it was also higher for the self-management group than for the knowledge-only group. The intervention was more effective for male students than females.
Pirzadeh, A. et al., (2020) [38] Iran, Isfahan	University	Quasi-experimental	*N* = 278 high school students Mean age is not described.	Two web-based intervention groups. One group received education through a website with tailored education strategies based on TTM. The second group only received general education by the same website but without tailored materials.	Measured before intervention and 6 months after: Stage of exercise behaviour change questionnaire. Processes of change questionnaire. Decision-making balance questionnaire. Exercise self-efficacy scale. International PA questionnaire short form.	Education on PA based on the website can be effective. The percentage of students with low, moderate, and severe levels of physical activity in the two intervention groups has increased significantly after the intervention. Participants showed significant progress during stages of change post-intervention and changes were greater in the group who was trained by the TTM.
Duan, Y. P. et al., (2017) [40] China, Central Region	University	Randomized controlled trial	*N* initial = 493 undergraduate students *N* post-intervention = 337 *N* 1-month follow-up = 142 Mean age of 19.2 years.	Web-based intervention modules target social–cognitive indicators for health behaviour change for Physical Activity and Fruit and Vegetable Intake (FVI) (information about risks and benefits, motivating intentions to change, identification of barriers, goal setting, development of action plans, coping plans and social support, providing tailored normative feedback).	PA by Chinese short version of the International Physical Activity Questionnaire (IPAQ-C). FVI in the past 7 days. Stages of behavioural change for PA and FVI. Social-cognitive indicators of behaviour change: positive and negative outcome expectancies for PA and FVI; self-efficacy for PA and FVI; action planning; coping planning; social support; intentions for PA and FVI; habit scale.	Students in the intervention group reported more FVI over time. Average FVI for the intervention group were all greater than the recommended amounts at the end of the 8-week intervention and the 1-month follow-up. In terms of PA behaviour, there was no significant interaction effect. Positive results on stage progression for the PA and FVI. All 6 tests revealed significant treatment effects on motivational, volitional, and distal indicators of PA and FVI over time.
Khalil, G. E. et al., (2017) [32] USA, Texas, Houston	School	Randomized controlled trial (2-arm single-blinded)	*N* = 101 adolescents Mean age of 13.4 years.	Two web-based intervention groups: One features interactivity and entertainment to engage adolescent users (text, animations, videos, task-oriented activities, two-dimensional environment to explore health information and make a virtual character). The second included the same health information but without any features of interactivity or entertainment.	Measured at baseline and follow-up: Intention to smoke using items adapted from the susceptibility to smoke scale.	The more participants considered intervention interactive and entertaining, the more they were probably going to show a reduction in their intention to smoke. Perceived interactivity had a more grounded relationship with the reduction in intention to smoke than perceived entertainment.
Castillo-Arcos Ldel, C. et al., (2016) [42] Mexico, Urban Mexico	School	Quasi-experimental (single-stage cluster sampling)	*N* = 193 participants Mean age of 15.8 years.	Multicomponent intervention: 6 online sessions + 2 face-to-face activities aimed at increasing levels of social competence and resilience about sexual behaviours.	Measured pre-and post-intervention: Self-reported risky sexual behaviours (defined as self-reporting unprotected sex, multiple concurrent sexual partners, and alcohol or drug use during sex). Resilience to risky sexual behaviour (defined as the ability to identify and practice strategies to avoid risky sexual behaviour).	The intervention was independently associated with improved self-reported resilience to risky sexual behaviours though not with a significant reduction in those behaviours in multivariate analyses. Participant age mediated the effect of the intervention on resilience, influencing the effectiveness of the intervention.
Doubova, S. V. et al., (2017) [41] Mexico, Mexico City	School	Quasi-experimental (field trial)	*N* = 833 adolescents Mean age is not described.	Multicomponent intervention: Educational sessions through a website displayed by two central characters + class discussions Main topics: dating, courtship, sexual relationships, misconceptions and myths about gender roles and sexual relationships, partner abuse, STIs, early pregnancy, self-esteem, safe sex, use of condoms and condom negotiation.	Measured at baseline, at the end of the four educational sessions (first month), and the end of the follow-up period (fourth month): Knowledge of STIs. Multidimensional Condom Attitudes Scale measuring attitudes regarding condom use. Self-efficacy toward consistent condom use.	The intervention had a positive effect on improving adolescents’ knowledge of STIs, attitudes and self-efficacy toward consistent condom use. In the intervention group, the average knowledge of STIs increased by 30 points compared to the control group. An increase in positive attitudes and self-efficacy toward consistent condom use was also observed more often in the intervention group.
Brown, K. E. et al., (2018) [36] United Kingdom (UK), Midlands	Clinical (sexual health service)	Pilot randomized controlled trial (two-armed parallel-group)	*N* initial = 88 integrated sexual health service attendees *N* follow-up = 67 Mean age of 20.0 years.	Multicomponent intervention: brief tailored web-based programme + paper-based action planning card. Content about contraceptive pills and/or condoms use using characters with audio to take the user through the process of identifying environmental cues to key target behaviours and planning to perform those behaviours when the environmental cue is present.	Measured at baseline and 3-month follow-up: Self-reported contraceptive pill or condom “mishaps” in the past 3 months. Contraceptive pill or condom use intention. Attitude toward contraceptive pill or condom use. Perceived behavioural control over pill or condom use. Subjective norm relating to pill or condom use. Trait self-control.	The intervention supported pill and condom users to produce quality plans since potential improvements were identified. Bivariate correlations suggest that perceived behavioural control may have a role over method use within intervention content. Additionally, having greater levels of trait self-control may negatively affect plan quality. The study suggests early indications that the intervention could reduce the number of mishaps of intervention participants.
Widman, L. et al., (2018) [33] USA, South-eastern	School	Randomized Controlled Trial	*N* = 222 tenth-grade girls Mean age of 15.2 years.	Interactive, skills-focused web-based intervention. The intervention includes 5 modules about safer sex motivation, HIV and other STDs, sexual norms and attitudes, safer sex self-efficacy, sexual communication skills that can be completed on a computer, tablet, or smartphone device. Each module used audio and video clips, tips from other adolescents, interactive games and quizzes, infographics, and skill-building exercises with self-feedback given in real-time).	Measured at pre-test, post-test and 4-month follow-up: Behavioural assessment of sexual assertiveness skills (at refusing unwanted sexual activity and negotiating condom use). Self-reported sexual assertiveness by Multidimensional Sexual Self Concept Scale. Knowledge regarding HIV and other STDs. Intentions to use condoms and to communicate about sex with items from the AIDS Risk Behaviour Assessment. Sexual Self-Efficacy from self-efficacy for HIV prevention scale.	Immediately post-test, the intervention group showed better sexual assertiveness skills measured with a behavioural task, higher self-reported assertiveness, intentions to communicate about sexual health, knowledge regarding HIV and other STDs, safer sex norms and attitudes, and condom self-efficacy compared with the control condition. At a 4-month follow-up, group differences remained in knowledge regarding HIV and other STDs, condom attitudes, and condom self-efficacy.
Arnaud, N. et al., (2016) [35] European countries (Sweden, Germany, Belgium, and the Czech Republic)	Online	Randomized controlled trial (Two-armed multisite)	*N* initial = 1449 adolescents (Convenience sample) *N* follow-up = 211 Mean age of 16.8 years.	Interactive web-based system to generate individually tailored content. Generated information in small units using text and graphics and referred to previous participants’ statements.	Measured at baseline and 3-month follow-up: Self-reported drinking index (drinking frequency, frequency of binge drinking, and typical quantity of drinks) using AUDIT-C screening tool.	Self-reported risky drinking as measured by a drinking index was significantly reduced for participants in the intervention group. Statistically significant mean differences at follow-up in favour of the intervention were found for drinking frequency and binge drinking frequency but not for quantity when missing follow-up data were not imputed. In contrast, analyses using an EM-imputed dataset revealed drinking quantity as the only significant secondary effect.
Norman, P. et al., (2018) [37] UK, large city	University	Randomized controlled trial (full-factorial design)	*N* initial = 2,951 students before starting university *N* post-intervention = 2681 Mean age of 18.8 years.	Brief online intervention combining self-affirmation x TPB-based messages x implementation intentions in a factorial design.	Measured at baseline, 1-week, 1-month and 6-month follow-up: Self-reported alcohol intake (total number of units consumed and number of binge drinking sessions/week). Hazardous and harmful patterns of alcohol use from 10-item AUDIT (only at 6-month follow-up). Cognitions about binge drinking (intention, affective attitude, cognitive attitude, subjective norms, descriptive norms, and perceived control) and extent of endorsement for the beliefs (Engaging in binge drinking at university would be fun; engaging in binge drinking at university would have a negative impact on my studies; my friends engaging in binge drinking would make my binge drinking at university more likely).	TPB-based messages had significant effects on reducing the quantity of alcohol consumed, frequency of binge drinking and harmful patterns of alcohol use over the first 6 months at university. Its effects did not diminish over time. The messages also had significant positive effects on intentions to binge drink, cognitive attitudes, subjective norms, descriptive norms, and self-efficacy, although some effects weakened over time. The effects on the quantity of alcohol and frequency of binge drinking were mediated by TPB variables with significant indirect effects through intention and self-efficacy. The effect sizes for the TPB-based messages on the quantity of alcohol consumed (d = 0.20) and the frequency of binge drinking (d = 0.17) were small. Messages were sufficiently relevant and persuasive to produce changes in behaviour without the need to form if-then plans. Non-significant effects were found for self-affirmation and forming implementation intentions.
Coughlin, L. N. et al., (2021) [34] USA, Michigan	Online	Pre/post-test design (Pilot study)	*N* = 39 participants Mean age of 20.7 years.	Mobile intervention with tailored messages and tips, inspirational images to reinforce content, web links to articles, or other web-based resources, based on users’ responses to daily and weekly surveys. The intervention included gamification through a virtual aquarium environment.	Measured at baseline and 1-month follow up: Concerning alcohol use (quantity and frequency of use, consequences of use, intention, importance confidence of change, perceived risk, reasons for use, and past month driving under influence of use).	Participants’ substance use declined over time, and those reporting using the app more often reported less substance use (including fewer days drinking alcohol, binge drinking, fewer consequences of use and episodes of driving after drinking) at the 1-month follow-up than those who reported using the app less often.
Doumas, D. M. et al., (2021) [28] USA, Northwest region	School	Randomized controlled trial	*N* = 311 high school seniors Mean age of 17.1 years old.	Online personalized normative feedback intervention via text, graphs, and video recordings. The program is intended to reduce risk factors for alcohol use and increase protective behaviours.	Measured at baseline, 30-day and 6-month follow-up: Weekly drinking quantity. Estimated peak blood alcohol concentration (eBAC). Self-reported peak alcohol volume. Classification of High-Risk vs. Low-Risk drinkers by participants’ report on the frequency of binge drinking in the past month.	The intervention effects were moderated by risk status, such that high-risk students in the intervention condition reported a greater reduction in alcohol use relative to students in the control condition. For weekly drinking quantity, intervention effects were limited to the baseline to 30-day follow-up period. Among high-risk students was found a significant decrease in weekly drinking in the intervention condition. However, intervention effects from baseline to the 6-month follow-up were not significant since the control condition also reported significant decreases in weekly drinking. For eBAC, intervention effects were evident at the 30-day follow-up and were sustained at the 6-month follow-up. Specifically, among high-risk students, we found a significant decrease in eBAC relative at the 30-day and 6-month follow-up. It is unclear why sustained intervention effects were found for eBAC but not for weekly drinking. Non-significant intervention effects for low-risk drinkers.

### 3.3. Recruitment and Participants

Our review encompasses data from 7616 participants, with 10 included studies having relatively small size samples (≤150 subjects per study’s condition or control) [28,30,31,32,33,34,36,38,39,42]. Sample sizes ranged from 20 subjects in the published work from Wilson, M. et al., (2017) [30] to almost 3000 participants in the study from Norman, P. et al., (2018) [37].

Data included in our review were collected from participants aged 10 to 24 years old, with an average mean age of 16.7, from all studies, except two of them who did not indicate the mean age of subjects [38,41]. The majority of the studies included older adolescents (aged > 14 years old) [28,30,33,35,37,38,41,42], only one was focused only on younger adolescents [39], two also encompassed younger adolescents (aged ≤ 14 years old) alongside older ones [31,32], and three also analysed emerging adults (aged < 25 years old) [34,36,40].

Concerning ethnic background, most interventions were tested in Caucasian participants [28,30,33,34,35,36,37], three focused on Asian participants [38,39,40], another three were implemented in Hispanic participants [31,41,42] and one mostly included both Hispanic (43.6%) and Afro-American participants (41.6%) [32].

There were more females than males in most studies [28,30,34,37,40,41,42], with two of the included studies only targeting girls [31,33], yet none were targeted only to male participants. In three studies, slightly more than half of the sample were men [32,35,39]. No information about sex representativity was available in Brown, K. E. et al., (2018) [36] and Pirzadeh, A. et al., (2020) [38].

In most of the studies, recruitment was achieved through educational institutions using institution-wide announcements, information sessions in lectures, classrooms, or after-school program meetings, and by sending emails and letters to participants and parents when applied [28,30,32,33,37,38,39,40,41,42]. Social media advertisements [34,35], announcements in health promotion sites, open access to the intervention’s website landing page [35], printed promotion materials distributed in public areas (such as schools, cafes, bars, stores, youth meetings and health-focused community events) [31,35], referencing by other participants [31] and using a brief verbal introduction and printed material presented by the staff to clinical attendees [36] were other recruitment strategies identified in the included studies.

These recruitment strategies resulted in non-probabilistic samples in all these trials, so results may not be generalized to out-of-sample contexts.

In eight studies, financial rewards, gift cards, giveaway items and prize draws were used as incentives for retention [28,30,32,33,34,35,37,42].

Only three studies had 6 months of follow up [28,37,38]. The others had a shorter length of study follow-up (<6 months) [31,33,34,35,36,39,40,41] and three of them did not include follow-up measurements aside from the moment immediately post-intervention [30,32,42].

### 3.4. Web-Based Interventions

A variety of web-based interventions were evaluated in the included articles, from brief online interventions based on text messaging delivered through e-mail with multimedia content links [37] or wearable digital tracking devices to record data and provide feedback on progress using an internet-based platform [30], to websites using narrative and animation to deliver content and challenges into a real-life context combined with a geographical information system to record progress [39].

Nearly half of them were at least somewhat tailored [33,34,35,36,38,40] and the degree of customization was also variable, ranging from interactive systems designed to generate individually tailored content matching participants’ response choices [35,36] to intervention elements that were consistent with the participants’ level of motivation/readiness to change and personalized reports on the participants’ progress [29] according to their questionnaire responses.

From among our 14 included articles, 10 interventions were exclusively internet-based and used the web to deliver all intervention components including the online data collection [26,29,32,33,34,36,37,38,39]. Most were delivered through a website and one of them was presented as a mobile application to create a gamification environment, a data collection field and shared affiliation links to other web-based resources [30]. However, in the other four studies, the web-based component was merely one element of a multicomponent intervention, such as using a wearable digital tracking device to record progress in an internet-based platform combined with workshops, lectures, and goal setting counselling face-to-face with professionals [31] or a brief tailored web-based programme with paper-based action planning cards [35], or to support online educational sessions with face-to-face sessions [41] or class discussions [40].

All our identified trials were health promotion interventions and included content to promote behavioural change on a range of topics, such as dietary patterns and healthy eating, physical activity, alcohol and tobacco use, and sexual behaviour.

### 3.5. Behaviour Change Theories and Techniques

All the included studies were theory-based interventions. Some of them were constructed based on only one theory or model, such as the Transtheoretical Model/Stages of Change [37], Operant Conditioning Theory [30], Information–Motivation–Behavioural Skills Model [40], Conceptual Framework of Adolescent Sexual Resilience [41], Theory of Motivational Interviewing [34], Health Action Process Approach [39], and the Self-efficacy Theory as a subset of the Social Cognitive Theory [31]. In contrast, other studies relied on more than one theoretical model, combining, namely: the Social Cognitive Theory with the Transtheoretical/Stages of Change Model [29], the Experiential Learning Theory and the Extended Elaboration Likelihood Model [32], the Theory of Planned Behaviour with the Health Action Process Approach [35] or with Self-affirmation and Implementation Intentions [36], the Social Cognitive Theory and the Health Belief Model [38], the Social Norming Theory with Motivational Enhancement models [26], as well as the Reasoned Action Model and Fuzzy Trace Theory with multiple others psychological and health behaviour change techniques [33].

### 3.6. Effectiveness of the Web-Based Interventions

Among the 14 included studies, three used differences between pre and post-test assessment [30,31,34] to document their effectiveness, while the remaining 11 based their findings on differences from intervention group to control groups, using active and non-active-control groups. Namely, six studies used a control group as assessment-only [28,35,36,37,38,40]; three studies used a non-web-based educational intervention as the control group, with one regarding the study outcome [41], while the other two were about generic health themes other than the one being studied [39,42]; the last two were web-based interventions, where one was about an unrelated health theme [33] and the other used a website with only written content and without interactivity or entertainment features [32].

Thirteen of the fourteen studied interventions revealed significant positive findings that support web-based intervention effectiveness in promoting health behaviour change, namely in improving motivation [30] and the practice of physical activity [31,38,39] as well as positive changes in weight, fitness and cardiovascular measurements [30]; in decreasing self-reported problematic alcohol use [28,34,35,37] and alcohol-related consequences [34]; in improving sex norms and attitudes, self-efficacy, self-reported sexual assertiveness skills, intentions to communicate about sexual health, knowledge concerning to sexually transmitted diseases and condom use [33,41] and to mitigate the numbers of mishaps in pill and condom use [36]; in reducing intention to smoke in non-smokers [32]; and in increasing fruit and vegetable intake [40].

Although the study from Castillo-Arcos Ldel, C. et al., (2016) had observed a crude reduction in risky sexual behaviours in the intervention group, they were not able to show a significant reduction in those behaviours using multivariate analyses since unexpected effects in pre and post-test scores occurred in the control group. It is important to note that the control group was subjected to the visualization of an educational video aimed to improve general health status, focusing on unhealthy food habits, mental health disorders, drug use, violence, and accidents [42].

### 3.7. Other Outcomes

The most frequent non-health-related outcome measured in the included studies was acceptability [30,31,34,36,39,41], but feasibility [30,31,36], engagement [32,34], adherence [30,31] and usability [30] were also evaluated in some studies.

The main aim of some of the studies was even to test feasibility and acceptability, being the evaluation of potential efficacy, a secondary objective given the pilot nature of those trials [30,31,34,36].

In these studies, the interventions overall proved to have reasonable levels of acceptability (ranging from moderate [31,36] to good [30,34,39,41]) and good feasibility [30,31,36,42]. As positive features, web-based interventions were classified by participants as easy to use [34], interactive and entertaining [32]. The time demanded to accomplish proposed activities [41], technical problems [34] and high drop-out rates [35,40] were negative aspects of some of these interventions.

### 3.8. Risk of Bias Assessment

The critical appraisal of individual studies performed using the Effective Public Health Practice Project—Quality Assessment Tool for Quantitative Studies (EPHPP) [23] for selection bias, study design, confounders, blinding, data collection methods, withdrawals and drop-outs are described in Table 3. Overall, the EPHPP tool showed the low quality of study methodology since 12 studies were classified as weak [28,30,31,33,34,35,36,37,38,39,40,42] and two as moderate [32,41].

## 4. Discussion

### 4.1. Summary of Findings

Most previous systematic reviews about digital health interventions are limited to the self-management of clinical conditions or symptoms instead of focusing on health promotion [43,44,45,46,47] or try to understand only one major health outcome change such as nutrition-related behaviours [48,49,50,51], sedentary behaviours [52] and physical activity [53], depression and mental health [54], alcohol-related problems [55] and their target population is other than adolescents such as adults [56] and older adults [57].

Although one previous review and meta-analysis performed by Wantland, D. J. et al., (2004) had found substantial evidence that the use of web-based interventions could improve knowledge and/or behavioural change outcomes when compared with non-web-based interventions, that one focused on the general population [58].

Since we hypothesized that its effectiveness could be more relevant in a very digital-skilled population, such as young people, our review intended to evaluate the effectiveness of web-based interventions in health behaviour change in adolescents. Moreover, due to the rapid growth of the technological field, an update focused on the most recent literature was justified.

As well as the work from Wantland, D. J. et al., (2004) [58], our systematic review also showed positive effects of internet-based interventions to achieve health behaviour change, including increased motivation [30] and physical activity level [30,31,38,39], decreased harmful alcohol use and its consequences [28,34,35,37], improved attitudes, self-efficacy, assertiveness skills, intentions to communicate and knowledge concerning to sexual behaviours [33,36,41], decreased intention to smoke [32], and increased fruit and vegetable consumption [40].

However, these findings relied mostly on small sample sizes [28,30,31,32,33,34,36,38,39,42], non-probabilistic samples, and studies with a lower length of follow-up, very context-specific [28,30,31,32,33,34,35,36,37,38,39,40,41,42], which limits the generalizability of the results, as already described in the literature [59].

In addition, although all studies analysed presented statistically significant differences between groups (control vs. intervention or pre-test vs. post-test), not all evaluated the intervention effect size [31,36] and when they do, different analytic estimators were used, compromising the quantitative summary and interpretation of the dimension of the differences.

In addition to the widely used statistical significance, the use of effect size for each outcome should also be promoted, as it would allow for more detailed reading and interpretation of results [60].

Although our review suggests that web-based interventions are a promising approach to achieve health behaviour change, robust evidence from larger randomized controlled trials, from the population’s representative samples, with proven relevant effects, is still needed.

A wide range of web-based interventions was found. It is worth noticing that the more interactive and entertaining the intervention was, the higher was the participants’ retention in the study. It also increases the intervention’s acceptability and feasibility [32]. Overall, web-based interventions seem to have moderate to good acceptability and feasibility [30,31,34,36,39,41,42].

In contrast to the previous literature [61], we found that researchers are largely developing interventions based on theoretical frameworks and models, which has been shown to improve an intervention’s effectiveness [62]. Critical points to the quality of evidence seem to be mainly related to sampling issues, representativeness of populations and absence of blinding. We identified that some important information, which is required by risk assessment tools, is sometimes non-available or unclear. The previous literature also underlines that researchers should include more detailed descriptions of their web-based interventions to achieve improved research designs [63]. Therefore, examining in advance all the domains evaluated in these tools may help researchers to conduct more robust methodological studies with a higher quality of evidence.

Even though web-based interventions seem to be a promising approach in health behaviour change with positive acceptability among adolescents, robust evidence is still lacking. We keep making the same errors as in the past since we still lack results from larger randomized controlled trials from high-quality papers (lower risk of bias), with representative samples and testing the long-term maintenance of these health behaviour changes (time of follow-up > 6 months). These limitations are known by most of the authors, who refer to them in their papers [28,30,31,32,33,34,35,39,40,41]. Nonetheless, it is crucial to identify why they keep being reported repeatedly and, moreover, try to overcome them. It was also frequent that studies were being classified as pilot projects, highlighting the need to study the effectiveness of the intervention in other studies, but never publishing those randomized controlled trials with the sample size needed for the effect size wanted. This may partially be explained by the lack of financial resources as well as time availability. Planning, developing, ensuring internal testing and usability testing is very time-demanding and costly research since the development of a web-based intervention is often a back and forward process [15]. With limited funding and restricted time to accomplish the intervention, most researchers will fail in providing robust evidence. We suggest that more than developing new web-based interventions, researchers should unify their strengths and resources to largely test the existing intervention in different cultural contexts within different populations.

### 4.2. Limitations of This Review

The present review is intended to summarize the most relevant evidence available to assess the effectiveness of web-based interventions to promote health behaviour change in adolescents. We acknowledge that our selection of 2016 as the oldest reference comports the risk of excluding previous robust evidence. However, the recent global increment of the importance of the web in our daily life and the emergence of other digital innovative technologies justify our focus.

It is also a fact that in the last two years, the resources and efforts of the worldwide scientific community have focused on the issue of COVID-19, mitigating the investment in health-promotion interventions for children and adolescents, since the educational context where these interventions were often implemented has undergone critical adaptations. This may partially justify the reduced number of included papers despite the growing trend observed during the last years. Nevertheless, the mandatory lockdown reinforced the need to invest in technological health promotion strategies that may be implemented at a distance, large scale and with almost the same financial and human resources.

We are aware that, due to time restrictions, we left out other databases relevant to this topic, such as EMBASE, ERIC, B-on, and Emerald, among others. It has been suggested that piloting a sample of records through every review’s step, such as producing a “mini” review, could be used to effectively change the criteria in the data extraction table to ensure that the full review would include the most useful and relevant information, removing the need to re-visit the papers at a later phase [58]. Thus, we can consider this work as a “mini” systematic review since an update should be performed by running the search query in other relevant databases. In addition, searching in grey literature, which was not included in this review, could expand the number of eligible publications. Even though publication bias has been largely documented in the literature, it seems that this bias is increasing. It could be explained by the higher competition between researchers that tend to publish positive results rather than negative ones [59]. For this reason, searching the grey literature would give us a more realistic summary of evidence.

The main limitations of the present review include the combination of results from well-designed and less rigorously designed studies, their heterogeneity of studies in terms of setting, interventions, methods and outcome measures, and the lack of included records on sleep hygiene. Additionally, the absence of analysis by subgroups of health outcomes and active components of the behaviour change intervention may be considered a limitation. However, the low number of studies that used each health area and each behaviour change technique did not allow this analysis to be performed.

In addition to these limitations, to our knowledge, this is the first systematic review summarizing the effectiveness of web-based interventions in promoting a wide range of health behaviour changes focused specifically on adolescents. Important findings were highlighted to help researchers to reach high-quality evidence in the development and evaluation of web-based interventions. Authors should discuss the results and how they can be interpreted from the perspective of previous studies and of the working hypotheses. The findings and their implications should be discussed in the broadest context possible. Future research directions may also be highlighted.

## 5. Conclusions

Our findings support that web-based interventions significantly contribute to achieving health behaviour change among adolescents, regarding physical activity, eating habits, tobacco and alcohol use and sexual behaviour, with reasonable levels of acceptability and feasibility. Additionally, more evidence is needed to prove their effectiveness in long-term maintenance, since there are few studies with follow-up assessments longer than 6 months. As shown by the critical assessment of the risk of bias, these findings are of low-quality evidence, so it is urgent to test these web-based interventions in larger randomized controlled trials, within probabilistic samples, ideally in single or double-blinded design and testing the long-term maintenance of these health behaviour changes (time of follow-up > 6 months).

## Figures and Tables

**Table 1:** Quality assessment components and ratings for EPHPP instruments reproduced from Thomas BH, Ciliska D, Dobbins M, Micucci S. A process for systematically reviewing the literature: providing the research evidence for public health nursing interventions. Worldviews Evid Based Nurs. 2004. [23], with permission from John Wiley and Sons, Copyright © 2004 (License number 5266150531479 obtained on 11 March 2022).

Components	Strong	Moderate	Weak
Selection bias	Very likely to be representative of the target population and greater than 80% participation rate	Somewhat likely to be representative of the target population and 60–79% participation rate	All other responses or not stated
Study design	RCT and CCT	Cohort analytic, case–control, cohort. Or an interrupted time series	All other designs or not stated
Confounders	Controlled for at least 80% of confounders	Controlled for 60–79% of confounders	Confounders not controlled for or not stated
Blinding	Blinding of outcome assessor and study participants to intervention status and/or research question	Blinding of either outcome assessor or study participants	Outcome assessor and study participants are aware of intervention status and/or research question
Data collection methods	Tools are valid and reliable	Tools are valid but reliability is not described	No evidence of validity or reliability
Withdrawals and drop-outs	Follow up rate >80% of participants	Follow-up rate of 60–79% of participants	Follow-up rate of <60% of participants or withdrawals and drop-outs not described

**Table 3:** Risk of Bias Assessment—EPHPP Assessment Tool for Quantitative Studies.

Author, Year	Section Rating						Global Rating
	Selection Bias	Study Design	Confounders	Blinding	Data Collection Methods	Withdrawals and Drop-Outs	
Doumas, D. M. et al., (2021) [28]	WEAK	STRONG	STRONG	WEAK	STRONG	MODERATE	WEAK
Wilson, M. et al., (2017) [30]	WEAK	MODERATE	STRONG	WEAK	STRONG	MODERATE	WEAK
Larsen, B. et al., (2018) [31]	WEAK	MODERATE	STRONG	WEAK	STRONG	STRONG	WEAK
Khalil, G. E. et al., (2017) [32]	WEAK	STRONG	STRONG	WEAK	WEAK	NOT APPLICABLE	WEAK
Widman, L. et al., (2018) [33]	MODERATE	STRONG	STRONG	WEAK	WEAK	STRONG	WEAK
Coughlin, L. N. et al., (2021) [34]	WEAK	MODERATE	STRONG	WEAK	STRONG	STRONG	WEAK
Arnaud, N. et al., (2016) [35]	WEAK	STRONG	STRONG	WEAK	STRONG	WEAK	WEAK
Brown, K. E. et al., (2018) [36]	MODERATE	STRONG	STRONG	WEAK	STRONG	STRONG	MODERATE
Norman, P. et al., (2018) [37]	WEAK	STRONG	STRONG	WEAK	STRONG	WEAK	WEAK
Pirzadeh, A. et al., (2020) [38]	WEAK	STRONG	WEAK	WEAK	STRONG	STRONG	WEAK
Huang, S. J. et al., (2019) [39]	MODERATE	STRONG	STRONG	WEAK	MODERATE	WEAK	WEAK
Duan, Y. P. et al., (2017) [40]	MODERATE	STRONG	WEAK	WEAK	STRONG	WEAK	WEAK
Doubova, S. V. et al., (2017) [41]	WEAK	STRONG	STRONG	MODERATE	STRONG	STRONG	MODERATE
Castillo-Arcos Ldel, C. et al., (2016) [42]	WEAK	STRONG	STRONG	WEAK	WEAK	MODERATE	WEAK

## Supplementary Materials

The following are available online at https://www.mdpi.com/article/10.3390/nu14061258/s1, Table S1: Search terms. Table S2: Excel spreadsheet with extracted data from included studies. Table S3: Data extracted about Recruitment and Participants. Table S4: Data extracted about web-based intervention and behaviour change theories. Table S5: Data extracted about secondary outcomes.
