# Supplementary material for: The Effectiveness of Web-Based Interventions to Promote Health Behaviour Change in Adolescents: A Systematic Review
Source: Nutrients. 2022 Mar 16;14(6):1258. doi: 10.3390/nu14061258 (PMC8954163; doi:10.3390/nu14061258)
Supplement: Supplementary file 1 [file nutrients-14-01258-s001.zip › nutrients-1598831-supplementary.pdf]

Supplementary Material

Table S1. Search terms.

		Search Terms
**Population**		teenagers OR adolescents OR "young people" OR teen OR youth
		AND
**Intervention**		"Internet-Based Intervention" OR "Web-Based Intervention" OR "online intervention" OR "digital intervention" OR "mobile intervention"
		AND
**Health outcomes**	**Main Outcome**	"health behaviour" OR "health behavior" OR "behaviour change" OR "behavior change" OR "health change" OR "health promotion" OR "lifestyle"
		OR
	• Physical activity	(exercise AND (increase OR start OR maintain*)) OR "physical activity"
		OR
	• Eating habits and weight control	weight OR BMI OR "five a day" OR diet OR nutrition OR "healthy eating" OR "fruits and vegetables" OR sugar OR salt
		OR
	• Smoking use	(smoking OR tobacco) AND (cessation OR stop* OR quit* OR reduc*)
		OR
	• Alcohol use	alcohol AND (reduc* OR limit* OR decreas* OR "cutting down" OR "cut down" OR "cut back" OR less* OR curb* OR abstain)
		OR
	• Quality of sleep	sleep OR "sleep duration" OR "sleep quality" OR insomnia OR "sleep hygiene"
		OR
	• Sexual Behaviour	sex OR sexual* OR "safe sex" OR "responsible sex"

Table S2. Excel spreadsheet with extracted data from included studies.

https://docs.google.com/spreadsheets/d/1PNR8J3vI5KUdm25UNryiy9TnQGl8SBBI/edit?usp=sharing&ouid=111354436685496275514&rtpof=true&sd=true.

Table S3. Data extracted about Recruitment and Participants.

**Author, year, country**	**Participants**	**Recruitment**	**Incentives**
Wilson, M., et al. (2017)<br><br>USA, North-western United States	N = 20 students (convenience sample)<br>• enrolled in 10 - 12 grade<br>• able to access a computer with Internet<br>• able to read/ understand English<br>• willing to attend an after-school activity ≥ once 1/ week<br>• initial BMI at/ or above the 85th percentile<br>Mean age of 16,8 years, 60% female and 85% white.	Recruited from one public high school on a first-come, first-served basis.	Modest rewards included movie tickets, 5 $ gift cards for shopping or healthy food activities, gym passes, 50 $ gift card at final of follow up.
Larsen, B., et al. (2018)<br><br>USA (San Diego, CA)	N = 21 Latina adolescents<br>• aged 12-18 years<br>• could read and write in English<br>• underactive (<90 min/week)<br>Mean age of 14,7 years, 100% female and 100% Hispanic.	Recruited from advertisements and presentations in health-focused community events, schools, church youth meetings and other public areas and referrals from other participants.	?
Huang, S. J., et al. (2019)<br><br>Taiwan, Taipei City	N initial = 617 students (only 524 completed the full experience: 88 in the intervention self-management group, 146 in the knowledge-only group, and 290 in the control group.)<br>Mean age of 11,4 years, 51% of boys and 49% were girls.	Recruited from six elementary schools in 3 districts.	?
Pirzadeh, A., et al. (2020)<br><br>Iran, Isfahan	N = 278 high school students<br>(94 in the tailored web-intervention group, 97 in the untailored web-intervention group and 87 in the control group)<br>(?) Mean age and sex distribution.	Recruitment through an educational institution.	?
Duan, Y. P., et al. (2017)<br><br>China, Central Region	N initial = 493 undergraduate students (270 in the intervention group and 223 in the control group)<br>N post intervention = 337<br>N 1-month follow-up = 142<br>Aged 15-24 years<br>The mean age in the intervention group was 19,0 years and 19,4 years in the control group. 40% male in the intervention group and 52,5% in the control group.	Recruited from one university in the central region of China.	?

Khalil, G. E., et al. (2017)<br><br>USA, Texas, Houston	N = 101 adolescents<br>• aged 12 - 18 years<br>• student in a middle school or high school<br>• non-smoker<br>Mean age of 13,4 years and 44% were female, and most participants were black/African American (42%) and Hispanic/Latino (44%).	Recruited from after-school programs.	A gift card and giveaway items to complete each survey.
Castillo-Arcos Ldel, C., et al. (2016)<br><br>Mexico, Urban Mexico	N = 193 participants (96 in the control group and 97 in the intervention group).<br>• aged 14–17<br>• not married or in a common-law cohabiting relationship<br>Mean age of 15,8 years in the experimental group and 15,7 in the control group. In the experimental group, 62% were female and 65% were in the control group.	Recruited from institution-wide announcements and information sessions in an educational institution.	Portable data storage device and a gift certificate.
Doubova, S. V., et al. (2017)<br><br>Mexico, Mexico City	N = 833 adolescents<br>(246 in the intervention group and 210 in the control group)<br>• aged 14 - 15 years of age<br>(?) Mean age. 53% in the intervention group and 58% in the control group were girls.	Recruited from two public secondary schools from the most socioeconomically disadvantaged neighbourhoods in Mexico City.	?
Brown, K. E., et al. (2018)<br><br>United Kingdom (UK), Midlands	N initial = 88 integrated sexual health service attendees<br>N follow-up = 67<br>• aged 14 - 24 years<br>• currently sexually active, or planning to be soon<br>• currently accessing or planning to access either one or both contraceptive pills and condoms<br>Mean age of 20,3 years at baseline and 19,6 at follow-up. Most of the participants were white (59% at baseline and 46% at follow-up). (?) sex distribution.	Recruited from a sexual health service by clinical staff.	?
Widman, L., et al. (2018)<br><br>USA, South-eastern	N = 222 tenth-grade girls (107 in the intervention group and 115 in the control group)<br>Mean age of 15.2 years. Participants were 38% White, 29% Hispanic and 25% Black.	Recruited from 4 rural, low-income high schools.	10$ for returning parent consent regardless of whether consent was granted, 10$ pretest, 30$ for intervention and posttest and 10$ follow-up.

Arnaud, N., et al. (2016)<br><br>European countries (Sweden, Germany, Belgium, and the Czech Republic)	N initial = 1449 adolescents (Convenience sample)<br>N follow-up = 211<br>• aged 16-18 years<br>• online access<br>• positive screening for at-risk substance use<br>The mean age of 16,8 years and 48% were women.	Recruited using online (open access WISEteens landing page, advertisement via social media, links on health promotion sites) and offline methods (print promotion materials distributed in public areas schools, cafes, bars, stores young events).	Held a prize draw for tablet computers.
Norman, P., et al. (2018)<br><br>UK, large city	N initial = 2,951 students before starting university<br>N post-intervention = 2,681<br>Mean age of 18,8 years. Most of the sample (54%) were women and (75%) were White.	Recruited through e-mail using institutional contacts from educational institutions.	Prize draw at each time point.
Coughlin, L. N., et al. (2021)<br><br>USA, Michigan	N = 39 participants<br>• aged 16-24 years<br>• screened positive for past-month binge drinking or recreational cannabis use<br>Mean age of 20,7 years and 62% female and most of sample 64% were not described as racial minorities.	Recruited from social media advertisements.	Monetary incentives through electronic gif cards.
Doumas, D. M., et al. (2021)<br><br>USA, Northwest region	N = 311 high school seniors<br>Mean age of 17,1 years old, 56% were females and 85% white.	Recruited from two urban high schools.	Incentives included 100$ deposited to teacher's school account for supplies, bagel and pizza party after follow-up if achieved 60% or greater parental consent return rate.

Table S4. Data extracted about web-based intervention and behaviour change theories.

Author, year, country	Aim(s)	Intervention name	Intervention description	Duration	Behaviour change theories
Wilson, M., et al. (2017)<br><br>USA, Northwestern United States	To examine feasibility and receptivity of overweight adolescents joining a community-based group fitness program and to test preliminary efficacy of the intervention.	Wellness Incentive to Health (WITH)	Multicomponent Intervention:<br>wearable digital tracking device using an Internet-based platform + group physical activities + nutrition group education/ individual counselling session on healthy eating + weekly goal-setting sessions.	12-week. Most scheduled program activities and goal-setting sessions lasted 1 to 2 h after school and on weekends.	Theoretical concepts of self-efficacy (Bandura, 1997) as a subset of Social Cognitive Theory
Larsen, B., et al. (2018)<br><br>USA (San Diego, CA)	To report the feasibility, acceptability, and potential efficacy of a theory-based, web-delivered intervention for Latina adolescents, focused on increasing moderate to vigorous PA.	Niñas Saludables	Website mobile phone friendly (tailored Internet-delivered activity manuals, computer-expert system tailored reports, activity tip sheets, and a guide of local activity resources).	All participants received access to the Niñas Saludables study website for 12 weeks.	Social Cognitive Theory and the Transtheoretical Model and other behavioural strategies (e.g.,. goal-setting, self-monitoring, and increasing social support).

Huang, S. J., et al. (2019)<br><br>Taiwan, Taipei City	To investigate the effectiveness of a theory-based, technology-integrated website in promoting the PA of schoolchildren.	Exercise Journey to the West	Two experimental groups:<br>One using a web-based exercise program applying a self-management strategy combined with geographical information system mapping function and using a narrative animated cartoon. And the other was knowledge-only using only the animated story.	8-week intervention, during computer education lessons at the participating schools (40 min on each occasion).	Social cognitive theory, as well as Health Belief Model
Pirzadeh, A., et al. (2020)<br><br>Iran, Isfahan	To examine the effects of a web-based intervention on the promotion of physical activity among adolescents using the TTM.	Salamat	Two web-based intervention groups.<br>One group received education through a website with tailored education strategies based on TTM.<br>The second group only received general education by the same website but without tailored materials.	?	Transtheoretical model (TTM)
Duan, Y. P., et al. (2017)<br><br>China, Central Region	To test the efficacy of a Web-based intervention compared with a control group condition to improve PA and FVI in Chinese university students.<br>To examine the effects on behaviours, stages of change movement for PA and FVI, social-cognitive indicators for PA and FVI, and perceived mental health outcomes.	?	Web-based intervention modules target social-cognitive indicators for health behaviour change for Physical Activity and Fruit and Vegetable Intake (information about risks and benefits, motivating intentions to change, identification of barriers, goal setting, development of action plans, coping plans and social support, providing tailored normative feedback).	8 weeks, 1 session per week, which lasted about 20 min.	Health Action Process Approach (HAPA) model
Khalil, G. E., et al. (2017)<br><br>USA, Texas, Houston	To evaluate the impact of a Web-based intervention on adolescents' intention to smoke, while considering the experience of interactivity and entertainment as predictors of reduced intention to smoke.<br>To test associations that link participation in a Web-based program to changes in a health outcome.	ASPIRE	Two Web-based intervention groups:<br>One features interactivity and entertainment to engage adolescent users (text, animations, videos, task-oriented activities, two-dimensional environment to explore health information and make a virtual character).<br>The second group included the same health information as the previous but without any features of interactivity or entertainment.	4 sessions, 40 min each, spread over 1 month	Interactivity and entertainment to change health outcomes as supported by the experiential learning theory (ELT) and the extended elaboration likelihood model (E-ELM)
Castillo-Arcos Ldel, C., et al. (2016)<br><br>Mexico, Urban Mexico	To evaluate the effect of an Internet-based intervention to reduce sexual risk behaviours and increase resilience to sexual risk behaviours among Mexican adolescents.	"Connect"	Multicomponent intervention:<br>6 online sessions + 2 face-to-face<br>Activities aimed at increasing levels of social competence and resilience about sexual behaviours.	8 sessions of 1h (6h online)	Conceptual framework of Adolescent Sexual Resilience
Doubova, S. V., et al. (2017)<br><br>Mexico, Mexico City	To evaluate the effect of an internet-based educational intervention to increase knowledge of STIs, attitudes and self-efficacy toward consistent condom use in Mexican adolescents.	?	Multicomponent intervention:<br>Educational sessions thought a website displayed by two central characters + class discussions<br>Main topics: dating, courtship, sexual relationship, misconceptions, and myths about gender roles and sexual relationships, partner abuse, STIs, early pregnancy, self-	Four weekly 1-h internet-based educational sessions. Internet sessions were followed by a 3-month follow-up period	The information-motivation-behavioural skills (IMB) model.

			esteem, safe sex, use of condoms and condom negotiation.	including six 30-min class discussions	
Brown, K. E., et al. (2018)<br><br>United Kingdom (UK), Midlands	To respond to the following research questions:<br>Do participants find the trial acceptable and is it feasible?<br>Does the web-based intervention support the production of good quality action plans?<br>Is there a relationship between trait self-control and quality of action plans?<br>Are there any changes in self-report contraceptive pill and condom use mishaps at three-month follow-up in each intervention arm?	?	Multicomponent intervention:<br>brief tailored web-based programme + paper-based action planning card.<br>Content about contraceptive pills and/or condoms use using characters with audio to take the user through the process of identifying environmental cues to key target behaviours and planning to perform those behaviours when the environmental cue is present.	Around 10-15 min	Theory of Planned Behaviour and Health Action Process Approach
Widman, L., et al. (2018)<br><br>USA, Southeastern	To evaluate the efficacy of an interactive, Web-based sexual health program for developing sexual assertiveness skills and enhancing sexual decision-making in adolescent girls.	HEART	Interactive, skills-focused web-based intervention.<br>The intervention includes 5 modules about safer sex motivation, HIV and other STDs, sexual norms and attitudes, safer sex self-efficacy, sexual communication skills that can be completed on a computer, tablet, or smartphone device.<br>Each module used audio and video clips, tips from other adolescents, interactive games and quizzes, infographics, and skill-building exercises with self-feedback given in real-time).	Approx. 45 MIN	Psychological and health behaviour change theories, including the Reasoned Action Model and Fuzzy Trace Theory
Arnaud, N., et al. (2016)<br><br>European countries (Sweden, Germany, Belgium, and the Czech Republic)	To evaluate the effectiveness of a targeted and fully automated Web-based brief motivational intervention with no face-to-face components on substance use among adolescents screened for at-risk substance	WISEteens	Interactive web-based system to generate individually tailored content. Generated information in small units using text and graphics and referred to previous participants' statements.	Range 5-30 min [mean 15.5 min (7.1)]	Theory of motivational interviewing (decisional balance, confidence and importance ruler, and provision of behavioural and regulatory strategies to resist peer pressure) and individual feedback techniques

Norman, P., et al. (2018)<br><br>UK, large city	To test if combining (1) messages that target key beliefs from the TPB that underlie binge drinking, (2) a self-affirmation manipulation to reduce defensive processing, and (3) implementation intentions (if-then plans to avoid binge drinking) reduces alcohol consumption in the first 6 months at university.	?	Brief online intervention combining self-affirmation x TPB-based messages x implementation intentions in a factorial design.	?	Theory of planned behaviour combined with self-affirmation and implementation intentions
Coughlin, L. N., et al. (2021)<br><br>USA, Michigan	To describe the iterative development and initial feasibility and acceptability testing of a mHealth smartphone app designed to reduce escalation in substance use.	MiSARA	Mobile intervention with tailored messages and tips, inspirational images to reinforce content, web links to articles, or other web-based resources, based on users' responses to daily and weekly surveys.<br>The intervention included gamification through a virtual aquarium environment.	30 days period	Operant conditioning theory (Motivational intervention framework, mindfulness, behavioural activation techniques)
Doumas, D. M., et al. (2021)<br><br>USA, Northwest region	To investigate the efficacy of the eCHECKUP TO GO on reducing alcohol use among high school seniors across the academic year.	eCHECKUP TO GO	Online personalized normative feedback intervention via text, graphs, and video recordings. The program is intended to reduce risk factors for alcohol use and increase protective behaviours.	30-min	Social norming theory and motivational enhancement models

Table S5. Data extracted about secondary.

Author, year, country	Secondary outcomes	
	Measures	Results
Wilson, M., et al. (2017)<br><br>USA, North-western United States	Computer Usability and Satisfaction Questionnaires at the final posttest.<br>Participation in each one of the 33 activities was also evaluated.	On average, participants attended 11.0 activities (SD = 7.2).<br>Eighteen participant evaluations (90%) were received at the program's end.<br>The mean value of the seven combined evaluation items was 2.3 (SD = 0.7), with 1 being the most favourable possible score.<br>Participants' records on the activity tracking Web site showed that they wore their digital device on average 31 days (SD = 21.8), amounting to 37% of a possible 84 total program days. Average days of use decreased over time. Calories consumed were recorded by only five participants.
Larsen, B., et al. (2018)<br><br>USA (San Diego, CA)	Acceptability was measured by satisfaction surveys.<br>Feasibility was determined by considering recruitment, retention, and adherence to the intervention.<br>The intervention was considered acceptable if at least 75% of participants indicated they were satisfied/very satisfied with the program and was considered feasible if at least 80% of participants	Acceptability - Overall satisfaction was moderate, with 72% of participants saying they were satisfied/very satisfied with the program.<br>Feasibility - 90% returned for the 12-week assessment.<br>Adherence was moderate. Approximately two-thirds (63%) of monthly questionnaires were completed. Only 42% reported<br>reading most/all of the individually tailored reports, and 42% reported reading at least half of the additional emailed tip sheets.

	were retained, defined as attending the 12-week assessment.	
Huang, S. J., et al. (2019)<br><br>Taiwan, Taipei City	Measured at baseline, immediately post and 3-month follow-up:<br>PA by the Chinese version of the Child/Adolescent Activity Log.<br>Exercise-related self-efficacy using a 5-item Exercise-Related-Self-Efficacy Scale.<br>Perceived benefit of PA using a self-developed 7-item Perceived-Benefit-of-Exercise Scale.	Effect on PA - students in both experimental groups was significantly more active than those in the control group, in addition, the self-management group scored higher than the knowledge-only group. The total amount of physical activity at the posttest reached a significant level. The self-management group was significantly more active than the knowledge-only and control groups.<br>Psychological determinants - a significantly higher level of perceived benefit ($F(2,502) = 3.84$, $p = .02$) and PA self-efficacy ($F(2,479) = 36.91$, $p = .00$) was discovered for the self-management group than for the knowledge-only and control groups, with the effect sizes being 0.015 and 0.135, respectively. However, no significant effect of perceived benefit was present 3 months later.<br>Further analysis revealed significant differences between all groups. Male students in the self-management group achieved a significantly higher amount of PA than those in the control group. However, no difference was discovered among the groups' female students. This demonstrated that the website intervention increased the amount of PA only in male students, with this increase sustained at 3 months later, but was not effective for the female students.
Pirzadeh, A., et al. (2020)<br><br>Iran, Isfahan	Nonapplicable	Nonapplicable
Duan, Y. P., et al. (2017)<br><br>China, Central Region	**Nonapplicable**	**Nonapplicable**
Khalil, G. E., et al. (2017)<br><br>USA, Texas, Houston	At follow-up, was measured perceived interactivity, perceived entertainment, and perceived credibility of the message.	No significant difference between the conditions regarding perceived credibility of message content, $F_{1,56}=3.67$, $P=.06$. Perceived credibility scores for both groups were found to be significantly greater than 3 on a 5-point Likert scale (ASPIRE: $t_{40}=4.38$, $P<.001$; control: $t_{42}=8.48$, $P<.001$), proving that both interventions were perceived to be credible sources of information related to smoking. Mixed-effects models showed that perceived interactivity and perceived entertainment independently worked as significant predictors of a decrease in intention to smoke (model 1: beta interactivity=−0.27, $P=.004$ and model 2: beta entertainment=−0.20, $P=.04$).
Brown, K. E., et al. (2018)<br><br>United Kingdom (UK), Midlands	Acceptability was measured by the % of participants who were willing to consent relative to the total eligible. Also, the number of participants willing to provide contact details and numbers who responded to provide follow-up measures vs. those lost to follow-up. Drop-out analyses assessed<br>whether participants lost to follow-up differed on key socio-demographic measures from those who remained.	Participation in the study appears to have been acceptable to clinic attendees. Of the 102 who reached the research room, 86.3 per cent agreed to take part and provide contact details for follow-up. Of those who completed baseline measures, provided contact details for follow-up, and were randomised to receive either the intervention or usual care, 76.1 per cent provided follow-up measures, representing relatively low attrition.<br>Chi-square analyses showed that dropping out vs. providing follow-up data was not significantly associated with sexual orientation (straight vs. not straight) of participants ($v2 = 1.158$; $df = 1$; $p = .282$); nor with ethnicity (white British vs. not white British) of participants ($v2 = 2.46$; $df = 1$; $p = .62$); nor with frequency of sexual activity (at least weekly vs. less than weekly sex) of participants ($v2 = .384$; $df = 1$; $p =.54$).

	Feasibility was assessed based on the acceptability measures above and assessment of the recruitment rate relative to the potential for recruitment based on the total number of clinic attendees during data collection periods.	
Widman, L., et al. (2018)<br><br>USA, South-eastern	Nonapplicable	Nonapplicable
Arnaud, N., et al. (2016)<br><br>European countries (Sweden, Germany, Belgium, and the Czech Republic)	Overall satisfaction<br>Drop-outs	The WISEteens intervention was pilot-tested in two steps and considering this pilot test, most adolescents were satisfied or totally satisfied with the system, design, comprehensibility, and intervention dialogue.<br>The intervention completion rate indicates acceptable user engagement in the “real world” and a good balance between the required amount of program exposure and adherence requirements of a single session. However, the dropout rate was high, and the authors consider it might be partly caused by invalid email addresses used by the participants and the fact that the system sent only one email reminder per participant.
Norman, P., et al. (2018)<br><br>UK, large city	Nonapplicable	Nonapplicable
Coughlin, L. N., et al. (2021)<br><br>USA, Michigan	Acceptability questions at follow-up. Participants were also asked about self-reported points earned in the app, which is a proxy measure for app engagement feasibility.	Acceptability - Most participants (79%) at least somewhat liked MiSARA, with almost all reporting that the design was appealing (92%). Most participants also felt it was not interactive enough (69%). Ratings for fun to use, interesting, and the aquarium was fun were modest. 75% of participants indicated the aquarium itself did not influence the time spent using the app. The content was viewed as age-appropriate (92%) and data collection via the app was acceptable (92% of participants agreed that they were comfortable with self-reporting, and 72% were comfortable with the app collecting passive data). Although the app was easy to use (87%) with clear instructions (90%), 23% of participants sometimes or regularly had technical problems.<br>Finally, most participants (74%) rated the app 3 or more stars.<br>Engagement Feasibility - Participants reported an average earning of 986.2 points (SD 688.0; range 0-2110; first quartile 324, third quartile 1591,<br>median 1163; n=34; 5 missing).<br>Alcohol use was the only baseline characteristic meaningfully associated with app engagement (estimate −10.5, SE 4.4; 95% CI −19.2 to −1.8), such that more alcohol use was associated with less app engagement.
Doumas, D. M., et al. (2021)<br><br>USA, Northwest region	Nonapplicable	Nonapplicable
